# Whole rumen metagenome sequencing allows classifying and predicting feed efficiency and intake levels in cattle

**DOI:** 10.1038/s41598-018-36673-w

**Published:** 2019-01-09

**Authors:** Beatriz Delgado, Alex Bach, Isabel Guasch, Carmen González, Guillermo Elcoso, Jennie E. Pryce, Oscar Gonzalez-Recio

**Affiliations:** 1 Escuela Técnica Superior de Ingeniería Agronómica, Alimentaria y de Biosistemas. UPM. Ciudad Universitaria s/n, 28040 Madrid, Spain; 2 Institució Catalana de Recerca i Estudis Avançants, ICREA, 08007 Barcelona, Spain; 3 Department of Ruminant Production, IRTA, 08140 Caldes de Montbui, Spain; 4 Blanca from the Pyrenees, Hostalets the Tost, 25795 Lleida Spain; 5 Departamento de Mejora Genética Animal, Instituto Nacional de Investigación y Tecnología Agraria y Alimentaria O.A., M.P, 28040 Madrid, Spain; 6 Bioscience Research Division, ECODEV, Bundoora, 3038 Australia

## Abstract

The current research was carried out to determine the associations between the rumen microbiota and traits related with feed efficiency in a Holstein cattle population (n = 30) using whole metagenome sequencing. Improving feed efficiency (FE) is important for a more sustainable livestock production. The variability for the efficiency of feed utilization in ruminants is partially controlled by the gastrointestinal microbiota. Modulating the microbiota composition can promote a more sustainable and efficient livestock. This study revealed that most efficient cows had larger relative abundance of *Bacteroidetes* (P = 0.041) and *Prevotella* (P = 0.003), while lower, but non-significant (P = 0.119), relative abundance of *Firmicutes*. *Methanobacteria* (P = 0.004) and *Methanobrevibacter* (P = 0.003) were also less abundant in the high-efficiency cows. A de novo metagenome assembly was carried out using de Bruijn graphs in MEGAHIT resulting in 496,375 contigs. An agnostic pre-selection of microbial contigs allowed high classification accuracy for FE and intake levels using hierarchical classification. These microbial contigs were also able to predict FE and intake levels with accuracy of 0.19 and 0.39, respectively, in an independent population (n = 31). Nonetheless, a larger potential accuracy up to 0.69 was foreseen in this study for datasets that allowed a larger statistical power. Enrichment analyses showed that genes within these contigs were mainly involved in fatty acids and cellulose degradation pathways. The findings indicated that there are differences between the microbiota compositions of high and low-efficiency animals both at the taxonomical and gene levels. These differences are even more evident in terms of intake levels. Some of these differences remain even between populations under different diets and environments, and can provide information on the feed utilization performance without information on the individual intake level.

## Introduction

The microbiome can be considered as a holobiont organism that populates different niches in mammals and interacts with the host, in most cases, in a symbiotic manner such as during the digestion of feed, or modulating the immune response^1,2^. Under certain dysbiosis, it can cause diseases and underperformance^3–7^. Recent research has proposed the microbiota as a proxy or phenotype to predict complex traits, such as body mass index in humans or feed efficiency in livestock animals^5,8–10^. Furthermore, links have been observed between the host genotype and the gastrointestinal microbial composition^8,10–13^, proving that the microbial communities that populate the individual digestive niches are not only dependent on environment and diet, but also on the host genotype. Microbiome research is gaining attention in livestock species, as it assists on understanding diseases and efficiency processes that occur in animals. In cattle in particular, the rumen microbiota is known to be associated with feed digestion and availability of nutrients for the host. In the year 2018, the rumen microbiome is estimated to be responsible for digesting around ten thousand million tons of cellulosic material worldwide to provide milk and meat for 7.6 billion people^14^. Depending on the microbiota composition, the input nutrients (feed) are transformed in an output product (milk) in a more or less efficient manner. Previous studies have related well-known taxonomical groups or community composition with feed efficiency or residual feed intake (RFI)^8,15,16^. Most of these studies used 16S rRNA sequencing as a description of the microbiota. This strategy provides limited information because reads must be aligned against incomplete databases that lack of specific rumen microbes. Besides, different taxonomical groups may be involved in similar functions, hiding true association at the gene function level when only looking at the taxonomical composition. There are previous international collaborations that aim to assemble the rumen metagenome in order to provide more comprehensive information on the microorganisms that populate the cow rumen^17–20^. However, few studies have associated feed efficiency traits to whole metagenome sequences, and their results have not yet been validated^5^. Feed efficiency is one of the most important characteristics in cattle due to its relationship with farm benefits, but also because its impact on securing food for a growing human population, decreased land use, or mitigation of greenhouse gas emissions. Feed efficiency has been traditionally improved via enhanced diets, and genetic selection to produce more milk per live weight. For instance, efficiency in dairy cattle has doubled in the last 50 years, even though feed efficiency has not been directly selected for. However, a recent study by Pryce *et al*.^21^ in the Australian cattle population showed that maintenance needs increased over time, as well as residual feed intake (a proxy for feed efficiency), leading to more money spent on feeding cows and a larger need of natural resources to sustain milk production. It also showed that indirect selection for efficiency led to impaired fertility, as the cows need to mobilize body reserves during the peak of lactation, generating a negative energy balance that preclude proper reproductive performance. Further research is necessary to develop strategies that perturb the microbiome in a more efficient manner, although these strategies rely thus far on recording individual feed intake in a small proportion of the population. Recording individual feed efficiency is extremely cumbersome and expensive, and it has become an important limitation to improve feed efficiency from genetic selection. The metagenomics era offers new opportunities to use microbiome composition to assess feed intake of an individual as well as its relationship with metabolic processes involved in the digestion, absorption, and utilization of nutrients.

The objective of this study was 1) to unveil potential associations between the rumen microbiota and traits related with feed efficiency in dairy cattle, and 2) to investigate the possibilities to use the metagenome as a proxy for these traits across individuals and different environments.

## Results and Discussion

Seventy Holstein lactating cows were kept under the same diet and management practices. Individual milk production, milk solid contents, dry matter intake (DMI) and body weight were recorded daily during a quarterly period. Cows were classified according to their feed efficiency (FE), calculated as milk production (kg/d) divided by feed consumption (kg/d). Then, cows belonging either to the high efficiency group (15 cows) or to the low efficiency group (15 cows) were selected. Ruminal samples were collected from each of these 30 cows using a stomach tube, and rumen metagenome sequences were obtained using shotgun sequencing.

### Taxonomical association with feed efficiency

**Figure 1:**
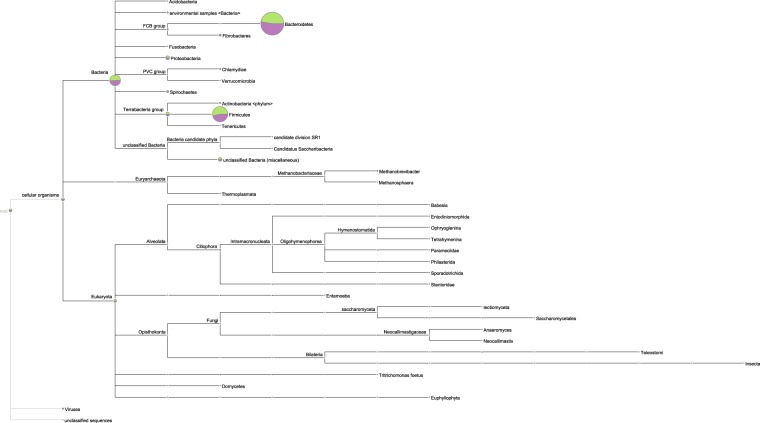
Taxonomy tree. Relative abundance of taxonomical groups in the high and low efficiency groups (Green = low efficiency, Purple = high efficiency).

Classification from MEGAN using the NCBI-nr database resulted in a poor classification of only 195 species from the rumen microbiota. Ninety six percent of the species belonged to the Bacteria kingdom, 1% to Archaea, 2.5% to Eukaryota, and 0.5% were unclassified. The bacteria community was mainly composed of *Firmicutes* and *Bacteroidetes*, with *Prevotella* being the most abundant genus (Fig. 1). This is in agreement with previous studies exploring the composition of the rumen microbiota in cattle, that reported that these phyla comprise around 90% of the 16S rRNA gene abundance^22–25^. As expected, there was inter-individual variation for the relative abundance at the specie level (Supplementary Figure S1).

**Figure 2:**
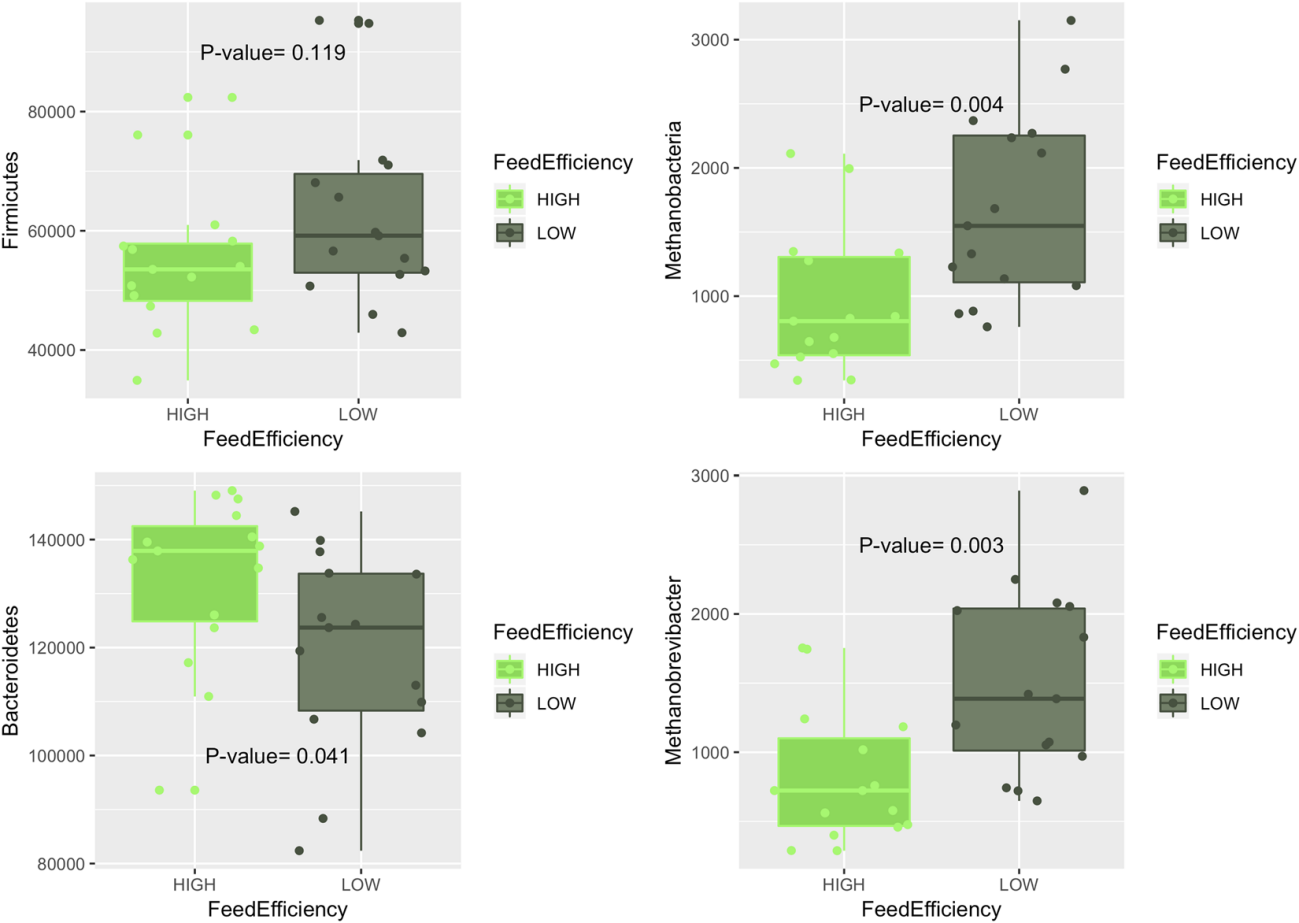
Association between selected OTUs and feed efficiency. Box plot and *P*-value of the efficiency group (low or high) from the logistic linear regression on the normalized abundance of *Firmicutes* (upper left), *Bacteroidetes* (bottom left), *Methanobacteria* (upper right) and *Methanobrevibacter* (bottom right).

More efficient individuals presented a larger relative abundance of *Bacteroidetes* (*P* = 0.041), and a lower, but not significant, relative abundance of *Firmicutes* (P = 0.119), as shown in Fig. 2. The most abundant genus in the *Bacteroidetes* group was *Prevotella*, which was also more abundant in the cows classified within the high-efficiency group (P = 0.003). The Archaea community was represented mainly by *Methanobacteria*, with *Methanobrevibacter* being the most abundant genus. Cows within the group of less efficient individuals presented larger abundance of *Methanobacteria* (P = 0.004) and *Methanobrevibacter* (P = 0.003) in their rumen microbiota (Fig. 2).

The ratio of *Firmicutes* to *Bacteroidetes* has been previously associated with obesity and metabolic diseases in both mice and humans^1,3,6^, with changes in fat composition in swine^26^, and also to milk fat yield in Holstein cattle^16^. A decreased amount of *Bacteroidetes* in the digestive microbiota has been also associated with increased lipidemia and fat deposition in different tissues in mice^6^, and with impaired feed conversion rate and residual feed intake^16^. Our results mirror in these previous studies as milk production requires a large amount of energy mobilization from tissues in high yielding cows^27^, in contrast a lower amount of *Bacteroidetes* in the rumen might redirect energy intake to an increased fat deposition at the expense of lowering milk production per unit of feed intake.

Methanogenic archaea deviate H_2_ and CO_2_ fermentation end-products from other microorganism to synthesise methane^28^, which represents a major sink for H_2_, and allows the fermentation of nutrients to carry on. *Methanobrevibacter* is the most abundant genus of *Methanobacteria* known in the rumen. The rumen microbiota of cows classified in the high-efficiency group had a lower (P = 0.003) abundance of this methanogen genus. Methane is not harnessed by the host, and is mainly exhaled through breath and eructation to the atmosphere. If methane production is lowered due to a reduction on *Methanobacteria* the entire fermentation in the rumen could be compromised because the oxidation reactions would not find a hydrogen sink. Alternatively, a reduced abundance of *Methanobacteria* within the high-efficiency group could be due to a lower proportion of H_2_ and CO_2_ being produced during the fermentation process by the rest of microbiota (*i*.*e*. carbohydrates are fermented to propionic acid, with no net loss of CO_2_ and thus lower substrate for *Methanobacteria* to produce CH_4_ and proliferate). The energy contained in a kg of methane has been estimated between 50 and 55.5 MJ, which could represent up to 12% of the total energy intake in dairy cows, which would be wasted in the form of a gas with 28 times greater green-house power than CO_2_. *Methanobrevibacter* have been associated with methane emissions in ruminants before^28–30^, together with the relative abundance of the *mcr* gene, which is specific of methanogenic microorganisms^31^. Ciliate protozoa have also been associated with methane production, as they are symbiotic and provide nutrients to methanogenic archaea. However, no differences were observed in the relative abundance of C*iliophora* (P = 0.422) between the high- and the low-efficiency groups in this study. The functions of protozoa are complex, as they are involved in many other feed fermentation and digestion processes and can even engulf bacteria and use them as protein and energy sources^28,32–34^. Furthermore, gene databases lack many protozoa representation, which may have led to biased determination of rumen protozoa in our samples or lack thereof.

### Metagenome association with feed efficiency

**Figure 3:**
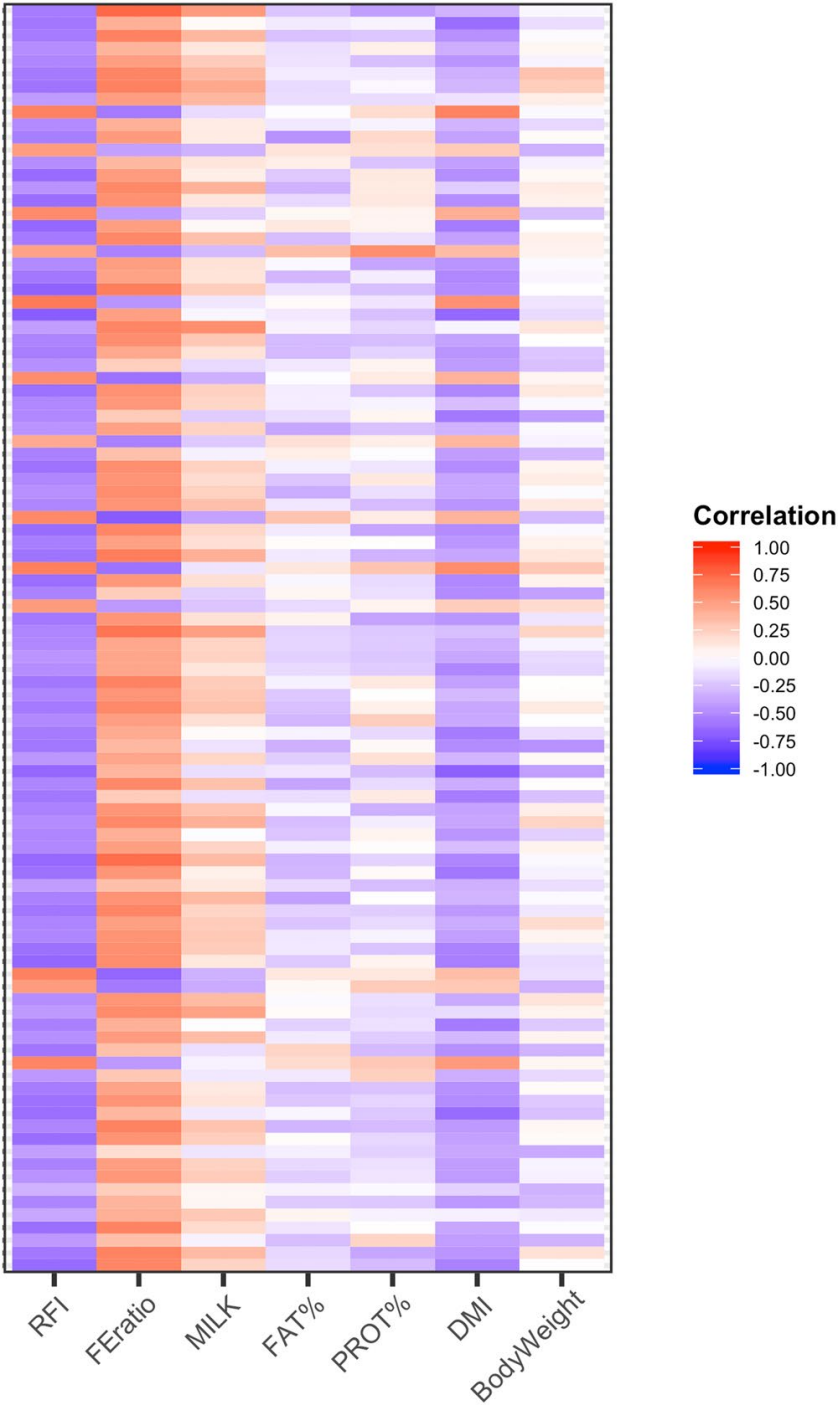
Correlation analyses. Spearman correlation between the 100 contigs with smaller p-value from metaGWAS analysis and feed efficiency related traits.

The metagenome assembly resulted in 496,375 contigs with an average length of 1,097 base pairs, and a maximum length of 42,126 bases. Ninety five per cent of the assembled contigs were mapped back by SALMON. After discarding contigs that appeared in less than 25% of the individuals, 175,969 contigs remained for further analyses. These contigs were used in an agnostic manner, with no assumption on their function or taxonomical classification. Filtering on information gain left 8,799 contigs for the metaGWAS^7^. Four hundred and twenty two contigs were statistically associated with feed efficiency. Average Spearman correlation between these contigs and FE was above 0.50 (in absolute value). A large correlation was also observed with RFI and DMI. Milk yield, milk solids, and body weight had weaker associations with these contigs. Fig. 3 shows the Spearman correlation between the 100 contigs with smaller *P*-value and feed efficiency related traits, sharper colours indicating stronger correlation. Larger (absolute) values were found for RFI and FE, averaging 0.55 and 0.50, respectively. First and third quartiles for the correlation between the contigs and FE (RFI) were −0.44 (0.50) and −0.59 (0.60). Correlation with DMI was also relevant, averaging 0.42. Productive traits (e.g., milk yield) showed weaker correlations (0.16–0.22) with the selected contigs. Also a weak association was found with body weight.

**Figure 4:**
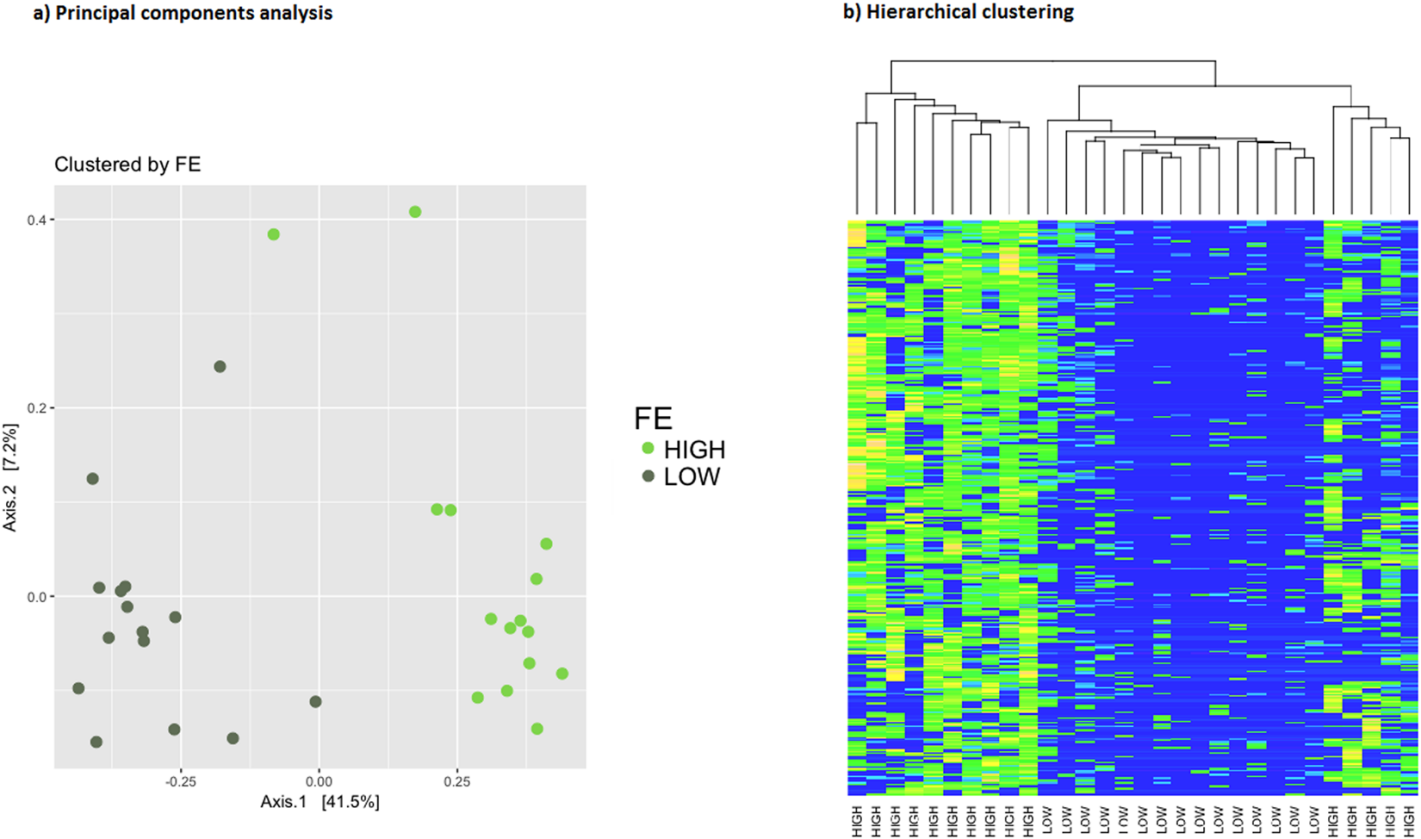
Cluster analysis of feed efficiency groups. The first two principal components of the CPM matrix for the selected contigs (left) show two clusters, one for the low group and one for the high group. The hierarchical clustering analysis (right) classifies animals in the high and low efficiency groups with an accuracy of 97%.

**Figure 5:**
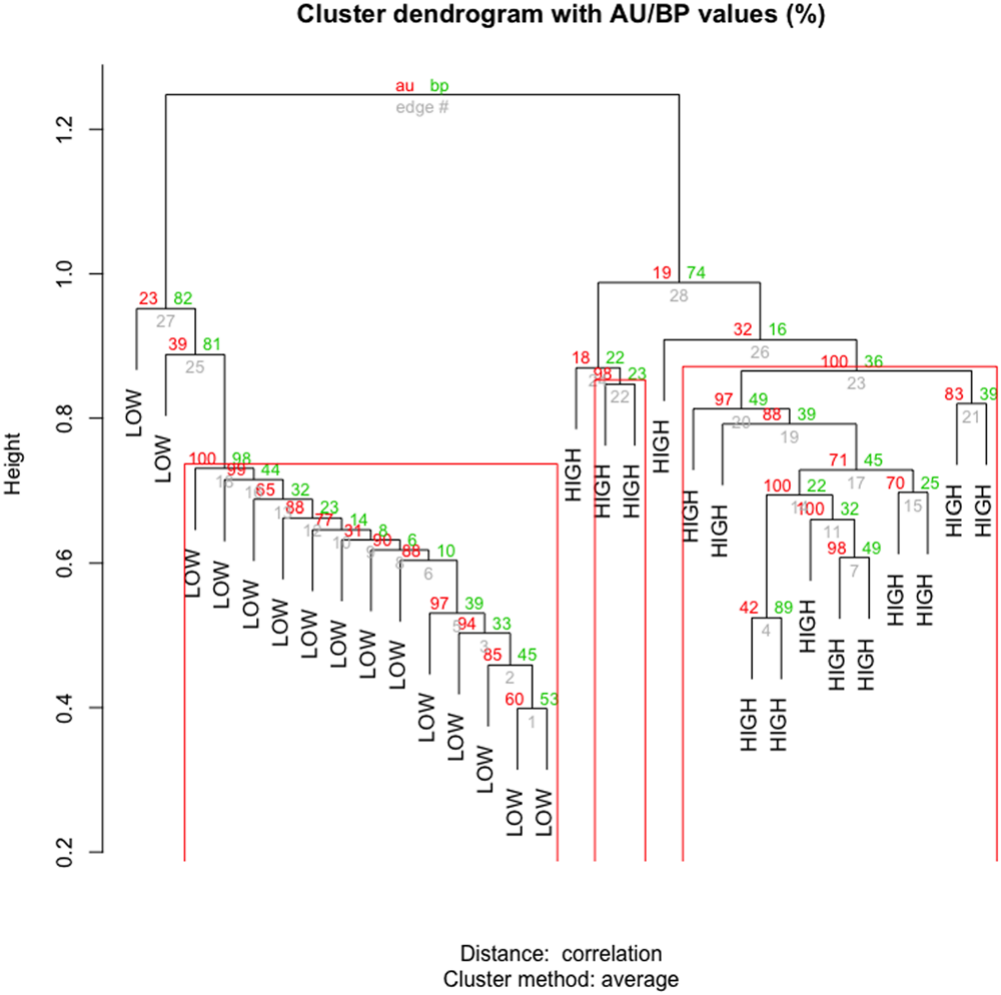
Significance of the hierarchical clustering. Hierarchical clustering based on selected contigs with bootstrap (bp) and approximately unbiased (au) values obtained from Pvclust with 1000 iterations.

The difference in the number of contigs per million (CPM) for these microbial genes allowed to separate high and low efficiency cows in a cluster analysis. Fig. 4 shows the principal components (4a) and cluster analysis (4b) from the selected contigs. There were two clear clusters that separated the cows based on their feed efficiency. The classification accuracy into either high or low efficiency was larger than 0.98 (Fig. 5). The uncertainty of these two clusters were computed via multiscale bootstrap resampling, resulting in *P*-values < 0.05. Hence, this is strong support against the null hypothesis, and we can safely conclude that these contigs are associated with FE.

**Figure 6:**
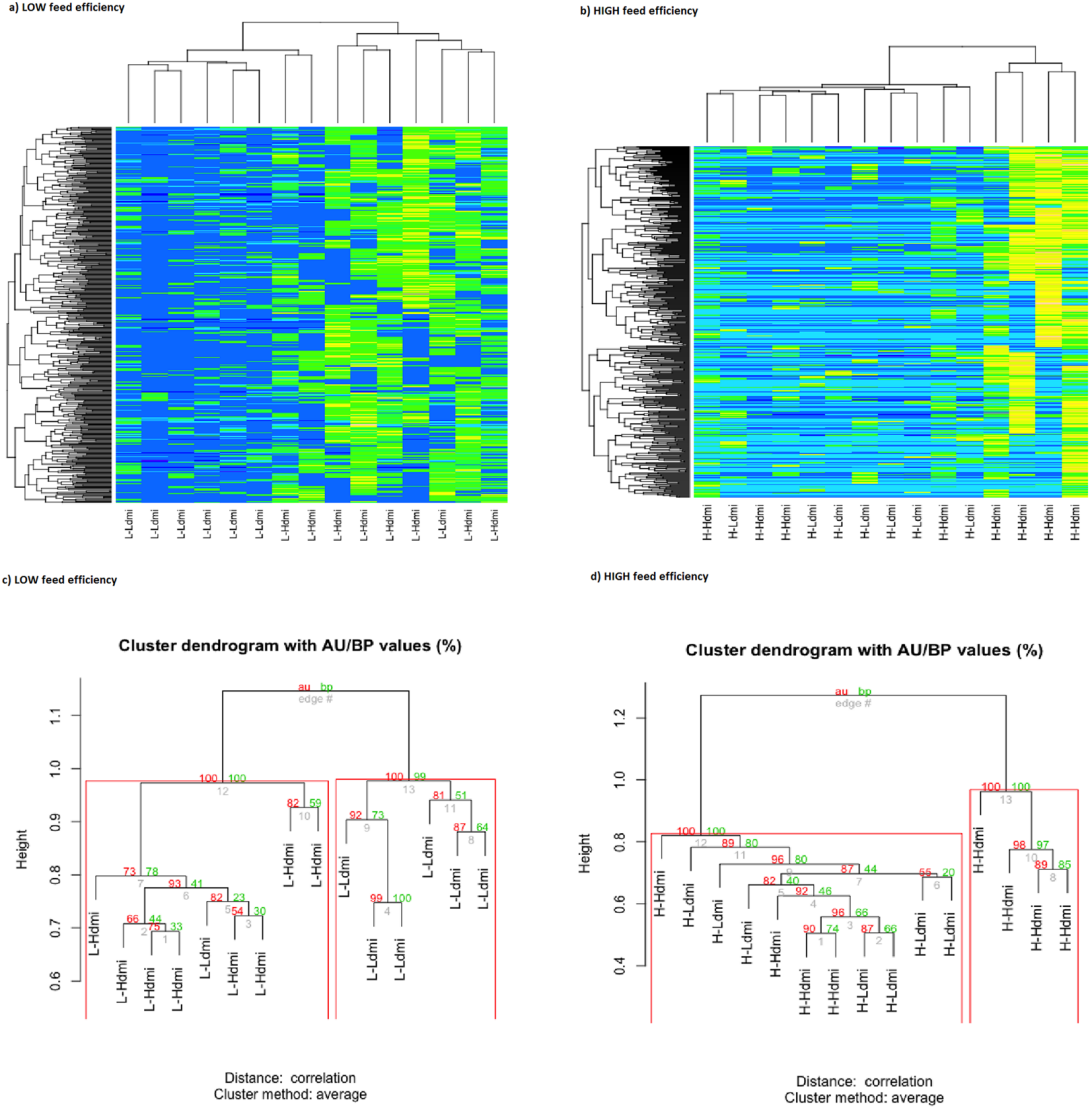
Clustering for dry matter intake. Hierarchical clustering for feed intake levels (top) within feed efficiency group, low (left) or high (right), based on selected contigs from metaGWAS analyses for dry matter intake. Bootstrap (bp) and approximately unbiased (au) values were generated using Pvclust with 1000 iterations (bottom). (each individual is assigned to any of the following groups: L-Ldmi = low feed efficiency and low dry matter intake; L-Hdmi = low feed efficiency and high dry matter intake; H-Ldmi = high feed efficiency and low dry matter intake; H-Hdmi = high feed efficiency and high dry matter intake).

In this study, FE had a larger correlation with milk yield (0.86) than with DMI (0.08). Hence, there is further interest on identifying high-producing and highly efficient animals with a low feed consumption. To accomplish this objective, a metaGWAS was conducted using DMI as phenotype, similarly as described above for FE. Here, 619 contigs were selected after statistical analyses. Hierarchical clustering also differentiated between cows with high and low DMI. The classification within FE group was highly accurate (Fig. 6a,b). Multiscale bootstrap resampling in the low FE group, resulted in 2 most probable clusters for high and low DMI, with *P*-values <0.05 and classification accuracy of 93% (Fig. 6c). The same uncertainty analyses in the high FE group resulted in two clusters (P < 0.05) (Fig. 6d) and classification accuracy of 67%. Interestingly, the cluster of high FE and high DMI comprised animals with the highest DMI phenotype (Supplementary Figure S2) within the most efficient animals in our data.

These clusterizations evidence that the efficiency of the individuals can be discriminated by the contigs in the rumen digesta under similar dietary and environmental conditions, and helps at determining what animals are more ravenous, indicating lower profitability in a farm at a similar production level.

Forty percent of the selected contigs presented homology with known genes. For instance, larger normalized number of CPM of the C1S69_178417 was correlated to larger feed efficiency. This contig presented homology with *fic* gene, which regulates growth and cell division^35^. Larger CPM of C1S69_141697 was also correlated with larger feed efficiency. This contig presented analogy with *araC* gene, which is a well-known regulator of the transport and catabolism of L-arabinose^36^. The contig C1S69_111990 presented homology with the *dus* gene, involved in many physiological processes included the glycolysis of galactose through the reduction of uridines^37^. The synthesis of vitamins A, K, and E through the *dxr* gene was also correlated with feed efficiency from the CPM of contig C1S69_487144. The *aroA* gene is involved in the synthesis of tyrosine^37^, which was correlated with feed efficiency (C1S69_248466).

**Figure 7:**
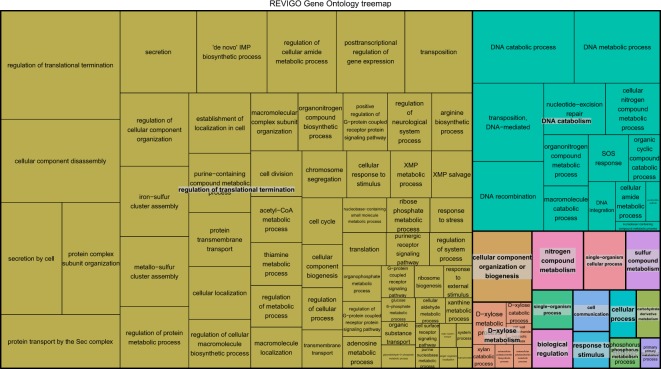
Tree Map. Two level hierarchy of gene ontology biological processes terms with enriched genes in the selected contigs associated to feed efficiency.

Figure 7 shows a two level hierarchy of gene ontology biological processes terms, which are known as TreeMap. Each rectangle is a single cluster representative, which are joined into superclusters of loosely related terms, and the size of rectangles reflect the enrichment *P*-value. We distinguish 15 super-clusters that point to processes related to gene expression and translation, cell life cycle, fatty acids and carbohydrates biosynthesis, fiber digestion from the feed and the release of nutrients that can be absorbed by the host (Supplementary Info I1).

**Figure 8:**
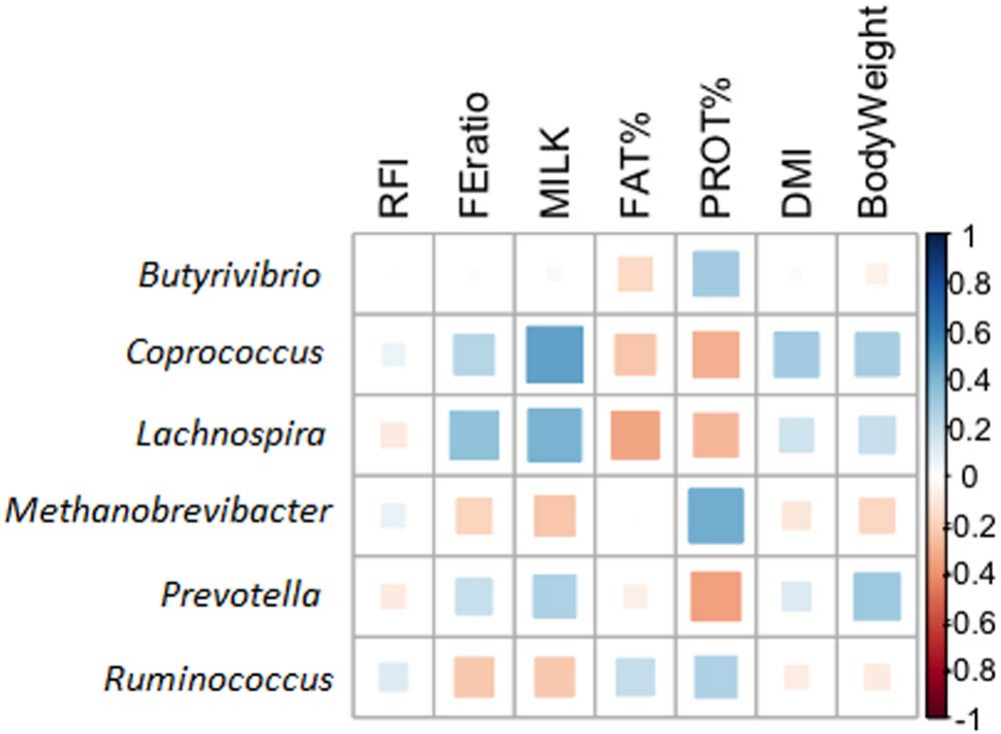
Genus correlated to feed efficiency. Spearman correlations between some of most studied genera and feed efficiency related traits.

These biological functions are typical of rumen microorganisms. For instance, *Butyrivibrio* and *Prevotella* genera are among the most abundant bacteria found in the rumen, and are involved in the metabolism of proteins and peptides. They break down protein and carbohydrates in feed, synthesize *de novo* peptides and use products of cellulose degradation from other cellulolytic bacteria as energy source^28,38,39^. They are also known to be involved in different steps of the ruminal biohydrogenation pathway of dietary unsaturated fatty acids^40^. Besides, *Butyrivibrio* bacteria are involved in the degradation of hemicellulose walls. Other bacteria are involved in energy-yielding mechanisms, such as *Ruminococcus*. They break down cellulose and hemicellulose and produce succinic acid as a major fermentation product together with acetic and formic acids, H_2_ and CO_2_. As discussed above, methanogenic archaea are also known to be negatively associated with FE^8,30,31^. Co-factors like F_430_ are essential during the methanogenesis^31,41,42^. Figure 8 shows the Spearman correlation between the relative abundance of these genera and feed efficiency related traits.

Ciliate protozoal communities are also abundant in the rumen^28,33^ (e.g. genera *Diplodinium*, *Dasytricha*, *Isostricyha*, *Eremoplastron*, *Entodinium* or *Trichostomatia*). They play a relevant role in digestion and fermentation of feed components, ruminal O_2_ scavenging, as well as stabilizing ruminal fermentation, engulf bacteria, and are symbiotic with methanogenic archaea^32,43^. They are considered fibrolytic microorganisms, that use soluble sugar, and are involved in many enzymatic activities participating in carbohydrate fermentation, producing acetate, butyrate, and lactate^28,34^, although no consistent results have been reported.

It must be pointed out that association does not imply causation, and clinical trials are necessary to confirm direct consequences from perturbing the microbiota composition. These findings might not be readily transferable to other populations and environmental conditions and other studies replicating these findings are neccesary.

### Independent validation

The large classification accuracy found in our data may be partially led by a common farm and diet environment. Different diets and environments are likely to change the microbiota composition, leading to different microbial gene pathways that, nonetheless, perform similarly in the digestion of feed and synthesis of nutrients that are absorbed and utilized by the animal. We used an independent data set from 31 Australian Holstein cows to determine to what extent the metagenome composition could predict the efficiency of individuals at feed utilization. The CPM for each cow were estimated with SALMON^44^ using the assembled metagenome from the BLANCA cows. Eighty five per cent of the assembled contigs were mapped back in the Australian dataset.

**Figure 9:**
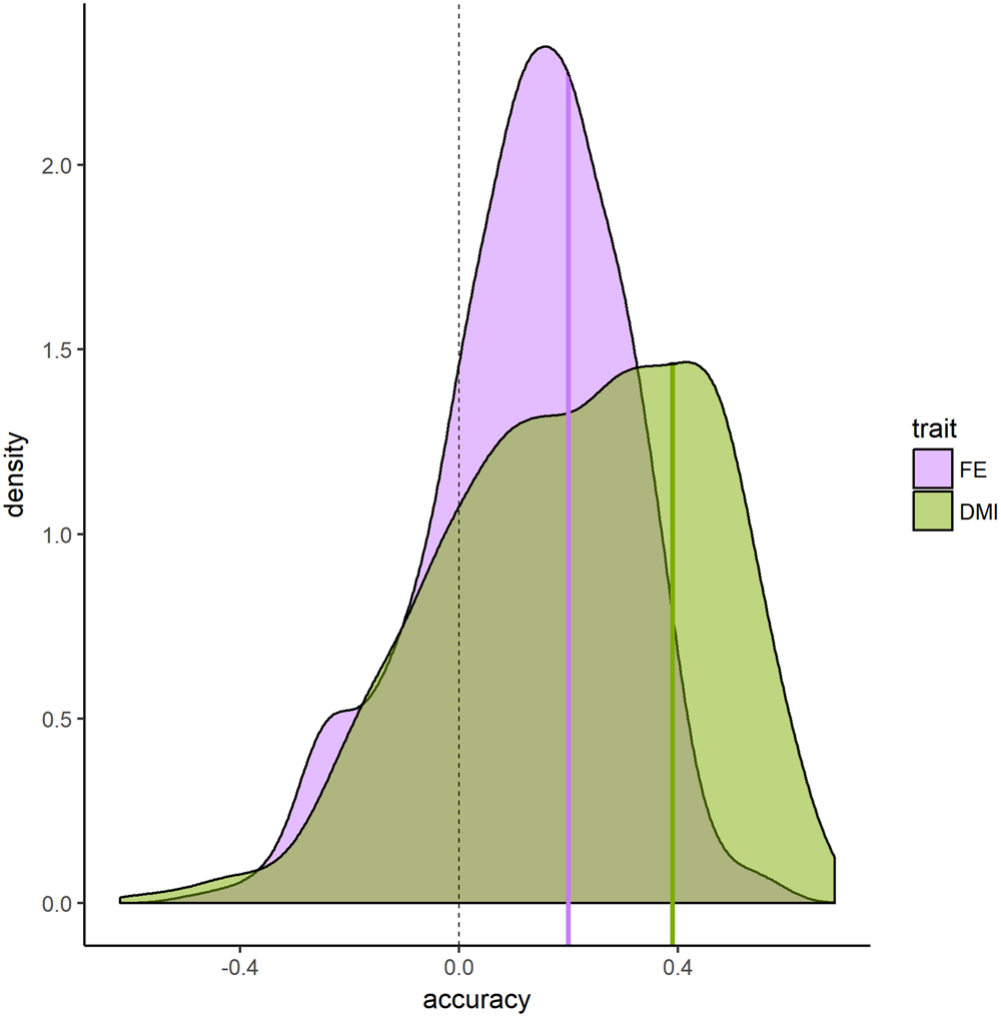
Validation accuracy. Density distribution of the Pearson correlation in the independent Australian population between the observed and predicted feed efficiency (purple) and dry matter intake (green) from 1000 iterations selecting 448 and 523 random contigs, respectively. Vertical lines show the respective Pearson correlations when the contigs with P-value < 0.025 (448 and 523 for feed efficiency and dry matter intake) selected in the BLANCA population were used to predict phenotypes in the Australian population.

Then, the BLANCA cows were used as the reference set with the previously selected contigs (either for FE or DMI) as explanatory variables. A low, but positive predictive accuracy was obtained in the Australian data set for both traits (0.19 and 0.39 for FE and DMI, respectively). The same analysis was performed selecting 422 and 619 random contigs for FE and DMI, respectively, equating the number of contigs to those with *P*-value <0.05 in the metaGWAS for each trait. In order to randomize the selection of contigs, 1,000 replicates were obtained with random sampling of the contigs at each replicate. It is expected that many contigs in the rumen microbiome provide some sort of information on feed efficiency, as there are many (if not all) processes related to feed digestion or microbiota composition somehow, which in turn can provide insights on the posterior utilization of nutrients by the host. Thus, it is expected that a random selection of contigs provides yet some information on the feed efficiency of the host. In this case, the average predictive accuracy in the validation data set was still positive (0.12 ± 0.01 and 0.21 ± 0.02 for FE and DMI, respectively), but lower than shown previously using the contigs with the lowest *P*-values. The 95% of the density distribution for the predictive accuracy after random selection of contigs ranged between −0.26 and 0.41 for FE, and between −0.26 and 0.60 for DMI (Fig. 9). The accuracies obtained using the selected contigs from the metaGWAS were in the 63^th^ and 73^th^ percentile of the distribution from random selection. These accuracies were above average, but they demonstrate limited statistical power from the metaGWAS given the relatively small sample size. There is room for improved predictive accuracy involving larger data sets and possibly more appropriated statistical methods.

Larger accuracies were obtained for DMI than for FE. This is expected as FE is a ratio trait composed from milk yield and DMI, and it dependson a larger number of factors such as feed composition, many biological processes during digestion and absorption of nutrients. On the other hand, the amount of intake is mainly dependent on appetite and rumen capacity. Nonetheless, as milk yield is routinely recorded in the majority of farms, there are encouraging opportunities to estimate FE using observed milk yield and predicted DMI from the metagenome information. Genomic selection has been proposed to tackle the lack of DMI records^21,45,46^. This consists on recording phenotypes and genotypes from a small reference population in experimental or highly technified farms, and use them to estimate SNP effects from this reference population. Then the merit for feed efficiency of any genotyped cow can be estimated based on such generated information^47^. Similarly, genomic selection can be applied to microbiota profiles if the microbiota of a sufficiently large number of individuals is evaluated.

The results from this study show that there are some microbial genes associated with feed efficiency related traits, which are conserved across different environments and hosts. Some of these microbial genes provide greater information about efficiency related traits, and a proper selection of those microbial genes may increase the predictive accuracy across different environments and populations. The results from the random selection of contigs show that similar predictive accuracies can be obtained with different combinations of contigs. The abundance of the microbial genes is expected to be redundant, with genes from different microbes performing similar functions. Further, large dependences of genes are expected in the microbiota (i.e., increasing the relative abundance of certain microbes might likely displace genes from other microbes in the same niche). Statistical methods accounting for redundancy between features are expected to select contigs in a better manner.

The prediction accuracy in this study was still low for direct application in the field, but it is yet encouraging given the reduced sample size, and it is expected that larger sample sizes will lead to larger accuracies. Further international collaborations building larger reference databases of metagenomes and phenotypes can largely increase the correlation shown here. Homogeneity in the protocols for sampling, and microbial DNA extraction can also lead to larger correlations. Some opportunities can be foreseen to predict traits related with feed efficiency with a sensible accuracy in farms that do not have the infrastructure or budget to measure individual feed intake.

## Conclusions

This study shows association between the microbiota and feed utilization and intake levels. Individuals with a larger relative abundance of *Bacteroidetes* were more efficient at feed utilization. Lower relative abundance of *Firmicutes* and metanogenic archaea were present in these individuals. More efficient individuals showed different metagenomes from those of less efficient animals. Similarly, more ravenous individuals modulated their metagenomes such that they can be clearly differentiated from those of individuals eating less. The microbial genes associated to feed utilization were involved in the digestion of non-fibre carbohydrates and fibre, as well as synthesis of fatty acids and protein, energy-yielding mechanism, and methane production. These microbial genes accurately classified individuals into the high or low groups for feed efficiency and feed intake level in this population. Furthermore, these genes predicted FE and intake level in an independent population, suggesting certain degree of similarity in the metagenome of more efficient and ravenous individuals even across populations, regardless of diet and environmental conditions. This predictive accuracy was limited in this study, although we showed that accuracies above 60% could be hypothetically obtained with increased statistical power.

Results from this study suggest that determining metagenome composition might assist as a phenotype proxy for feed efficiency in livestock species, but a large reference population need to be established with metagenome information that serves for the purpose of genomic selection on feed efficiency. It must imply lower costs than phenotyping for direct dry matter intake.

## Methods

This study was carried out in accordance with Spanish Royal Decree 53/2013 for the protection of animals used for experimental and other scientific purposes, and approved by the ethics committee of the *‘Institut de Reserca i Tecnologia Agroalimentàries’* of the Generalitat de Catalunya with number 9743.

### Data

Eighty Holstein cows from the *BLANCA from the Pyrenees* experimental farm were monitored during 2 weeks. All animals were under the same management routines, eat the same diet based on fescue, ryegrass and concentrate (Supplementary Table T1) and were in the same lactation stage (between 60–120 days post-partum). Daily milk yield, fat and protein contents, dry matter intake and body weight during the study period were averaged to obtain a single record per cow. Daily feed efficiency was calculated as the ratio between milk yield (kg) and dry matter intake (kg), and the average along the experimental trial was used as FE phenotype. Residual feed intake was calculated as the difference between observed and expected dry matter intake following^21^.

During the sampling, cows stood in individual stalls. Rumen content (approximately 50 mL) from each cow was sampled at day 7 using a stomach tube connected to a mechanical pumping unit, and collected in a sterilized container. Material was thoroughly washed between cows. All samples were frozen immediately after the extraction and then stored at −80 °C until analysis. The samples were thawed before the analyses, until they could be grin and homogenized in a blender. DNA extraction was performed using 250 µl from the homogenized samples with the “DNeasy Power Soil Kit” (QIAGEN, Valencia, CA, USA), and following the manufacturer’s instructions.

Genomic DNA concentrations and their purity were measured by spectrophotometry using a Nanodrop ND-1000 UV/Vis spectrophotometer (Nanodrop Technologies Inc., DE, USA). All DNA samples were diluted to a concentration of 5 ng/µl in a total volume of 15 µl/sample in a 96-well plate. Finally, all samples were sent to an external sequencing service (FISABIO, Valencia, Spain) where they performed the metagenome sequencing using Illumina MiSeq technology. In total, 9.07 Gb of forward and 9.30 Gb of reverse reads were obtained (Supplementary Table T2).

### Taxonomy Association Analysis

MEGAN6 Community Edition (CE)^48^ v6.11.5 was used for the taxonomic binning, avoiding a previous assembly step, using a weighted LCS algorithm. Reads obtained from Illumina were first aligned against the NCBI nonredundant (NCBI-nr) protein database (May 2017) using DIAMOND v0.913^49^. MEGAN provided the number of reads assigned to each group of a given taxonomic clade. Then, association analysis was performed using logistic regression^50,51^ on the relative abundances of the taxonomical phyla of *Firmicutes*, *Bacteroidetes*, and *Methanobacteria* and on the relative abundance of *Methanobrevibacter Spp.* The models included the number of calving (primiparous vs. multiparous), and feed efficiency group (‘HIGH’ or ‘LOW’) as fixed effects. A significance level of α = 0.05 was assumed.

### Metagenome ensemble

The 30 cows with extreme FE phenotype (15 larger and 15 lowest feed efficiency record) were selected to sequence the whole metagenome of their rumen digesta sample. Illumina libraries were prepared from the extracted DNA and sequenced on Illumina MiSeq v3 systems (2 × 300) by Fisabio (Valencia, Spain). Quality control of reads was performed, filtering reads out shorter than 50 bp and average Phred score <30 within a 20 bp window. De novo assemble of the metagenome was carried out using MEGAHIT^52^. A 30-metagenome co-assembly was carried out using options –k-min 21 –k-max 721 –k-step 10. The resulting assembly consisted of 496,375 contigs.

Then, microbial functional genes encoding for proteins (contigs) were identified using the KEGG genes database with PROKKA^53^. The annotation was performed with options –compliant –centre UoN –norrna –notrna –metagenome, and a bioproject was submitted to NCBI database with number PRJNA423102.

Quantification of contigs in each sample was performed with SALMON^44^. Only contigs appearing in at least 7 (out of 30) animals were selected for further analysis. The normalized number of CPM was used in downward analyses.

### metaGenome Wide Association Analyses

Pre-selection of contigs was performed using the information gain or entropy reduction criterion^51,54^. Information gain is the difference in entropy of a probability distribution before and after observing a variable contig, i.e., it measures how much uncertainty is reduced by observation of CPM. The entropy of the probability distribution of a discrete random variable Y is defined as:

$$H(\Pr (Y))=\,\mathrm{log}({\sigma }_{y\in A}\sqrt{2\pi \gamma }),$$

where ${\sigma }_{y\in A}$ is the standard deviation of the CPM for the respective contig in the sample, *γ* is the Euler constant, and the logarithm is on base 2 to mimic bits of information. The above pertains to a discrete distribution. Here, *Y* refers to the phenotype groups in the sample, and *A* is the set of all states that *Y* can take (‘HIGH’ or ‘LOW’).

For each contig, the data set was divided into 2 subsets corresponding to the 2 possible groups (‘HIGH’ or ‘LOW’). For each contig *k* there are ${N}_{k}^{High}$ individuals in the high class, and ${N}_{k}^{Low}$ individuals in the low class. The information gain for each contig *k* (*k* = 1, 2, …, 174, 247) was the change in entropy after observing the CPM, calculated as:

$$IG(conti{g}_{k})=H(\Pr ({\bf{Y}}))-\frac{1}{{N}_{k}^{High}+{N}_{k}^{Low}}\sum _{s=High,Low}({N}_{k}^{s}\,\mathrm{log}({\sigma }_{y\in s}\sqrt{2\pi \gamma })),$$

The contigs with largest information gain in the top 95 percentile were pre-selected for subsequent analyses. Note that the choice of the 95^th^ percentile was arbitrary, 99^th^ and 90^th^ percentiles were tested with no improvement.

### Single contig association analyses

Association analysis was performed using logistic regression^50,51^ of CPM observations from each contig on the observed response (‘HIGH’ or ‘LOW’, codified as 1 or 0, respectively). Selection of contigs for cluster analyses was performed on contigs that resulted significant at α = 0.05. The tool “microbiome” (Lahti L, Shetty S, Blake T and Salojarvi J (2012–2017). “microbiome R package.”) in R^55^ was used to perform association and cluster analyses.

Hierarchical clustering was then performed using the selected contigs in the Pvclust^56^ R statistical package using the average distance matrix method. Bootstrap (BP) and approximately unbiased (AU) values were computed.

### Enrichment analysis

The potential biological function of the genes located within the contigs on feed efficiency was detected using enrichment functional analyses with the DAVID^57^ tool in order to propose candidate genes that underlie the detected associations. The lists of genes within the selected contigs were uploaded in DAVID (http://david.abcc.ncifcrf.gov/). The results obtained by the default conditions (i.e., minimum 2 genes per term, EASE score ≤ 0.10) were downloaded. The terms of gene ontology biological processes that were enriched with the resulting genes (FDR <5%) were semantically summarized in clusters using REVIGO^58^ with default parameters and the whole UniProt database.

The annotations for each general group of annotation downloaded were from the KEGG pathways. The *P*-value and the Benjamini-Hochberg False Discovery Rate were used to determine significance of enrichment or overrepresentation of terms for each annotation (e.g., Gene Ontology Biological process).

### Independent validation

Beyond the knowledge on microbial genes affecting feed efficiency and its classification accuracy for animals under similar environments, the practical outcome of our work is to the metagenomic prediction of complex phenotypes related to feed efficiency across environments. The BLANCA population was used as reference set and an Australian population was used as validation set. A comprehensive description of data from the validation set can be found in^59^ and^5^. In summary, we used 16 and 15 cows from batches 1 and 2^59^ from the Victorian Department of Primary Industries Ellinbank Centre near Warragul, Victoria Australia (latitude 38 14’S, longitude 145 56’E). Cows received feed ad libitum, and were monitored for 32d and 37d, respectively. Individual intakes were determined using electronic monitoring of load cells under feed bins (Gallagher Animal Management Systems, Hamilton, New Zealand) and electronic identification of individual animals.

Rumen fluid was collected via a stomach tube. DNA was extracted using the PowerMax Soil DNA Isolation kit (MoBio) and sequenced on the HiSeq 2000 (Illumina). The sequences were filtered following the same criteria as above. The remaining data were aligned against the assembled methane from BLANCA cows using SALMON^44^ to calculate the CPM for each validation animal.

Using the CPM of selected contigs as predictors, we compute an estimator $\hat{{\boldsymbol{\beta }}}$ of the linear effects of CPM on the phenotypes as follows:

$${y}_{{\rm{i}}}={y}_{0}+{{\bf{x}}}_{i}{\boldsymbol{\beta }}+{e}_{{\rm{i}}}$$

where *y*_*i*_ is the phenotype (either FE or DMI) for animal *i* (*i* = 1, … *n*) in the reference data set, *y*_*o*_ is the adjusted population mean, **x**_*i*_ is the *i*th-row of the design matrix ${\bf{X}}=\{{x}_{ij}\}$ containing contig *j* CPM (*j* = 1, …, *p*) for individual *i*. The errors, *e*_*i*_, are assumed to be (identically and independent) normally distributed with unknown variance *σ*_*e*_. The vector of linear effects of contigs, $\hat{{\boldsymbol{\beta }}}\in {{\mathbb{R}}}^{p}$, was estimated using L1-penalized regression (LASSO)^60^. This corresponds to minimizing the objective function

$$\hat{{\rm{\beta }}}=\mathop{{\rm{\arg }}\,{\rm{\min }}}\limits_{\hat{{\boldsymbol{\beta }}}\in {{\mathbb{R}}}^{p}}\,{{\rm{O}}}_{\lambda }({\bf{y}},{\bf{X}};{\rm{\beta }}),\,{{\rm{O}}}_{\lambda }({\bf{y}},{\bf{X}};{\rm{\beta }})=\tfrac{1}{2}{\Vert {\bf{y}}-{\bf{X}}{\rm{\beta }}\Vert }^{2}+n{\rm{\lambda }}{\Vert {\rm{\beta }}\Vert }_{1},$$

where *λ* is a penalty (hyper-)parameter and the L_1_ norm is defined to be the sum of the absolute values of the coefficients ${\Vert {\rm{\beta }}\Vert }_{1}=\sum _{j=1}^{p}|{{\beta }}_{j}|$.

The first term is the standard ordinary least-squares loss function. The purpose of the second term is to regularize the regression problem by favouring sparse solutions with the nonzero coefficients shrunk toward 0 if a selected contig has no effect or this is already accounted for any other contig. Biasing the nonzero coefficients toward 0 reduces variance and improves the expected fit for small sample size, even for *n* ≪ *p*.

Note that a vector of estimator $\hat{{\boldsymbol{\beta }}}$ was obtained for each of the phenotypes (either FE or DMI). Then, FE and DMI were estimated in the Australian population as ${\hat{y}}_{i}^{\ast }={{\bf{x}}}_{i}^{\ast }\hat{{\boldsymbol{\beta }}}$, where ${\hat{y}}_{i}^{\ast }$ is the predicted phenotype for the *i*th individual in the testing set (*i* = 1, …, 31), and ${{\bf{x}}}_{i}^{\ast }$ is the corresponding *i*th-row of the contig CPM design for the validation set (${{\bf{X}}}^{\ast }$). The predictive accuracy was measured as the Pearson correlation between the estimated and observed phenotypes.

### Ethics approval and consent to participate

This study was carried out in accordance with Spanish Royal Decree 53/2013 for the protection of animals used for experimental and other scientific purposes, and approved by the ethics committee of the *‘Institut de Reserca i Tecnologia Agroalimentàries’* of the Generalitat de Catalunya with number 9743.

## Electronic supplementary material

Supplementary Info

## Data Availability

This Whole Genome Shotgun project has been deposited at DDBJ/ENA/GenBank under the accession REGX00000000. The version described in this paper is version REGX01000000.
