# Author Correction: Whole rumen metagenome sequencing allows classifying and predicting feed efficiency and intake levels in cattle

**DOI:** 10.1038/s41598-020-60003-8

**Published:** 2020-02-13

**Authors:** Beatriz Delgado, Alex Bach, Isabel Guasch, Carmen González, Guillermo Elcoso, Jennie E. Pryce, Oscar Gonzalez-Recio

**Affiliations:** 1 Escuela Técnica Superior de Ingeniería Agronómica, Alimentaria y de Biosistemas. UPM. Ciudad Universitaria s/n, 28040 Madrid, Spain; 2 Institució Catalana de Recerca i Estudis Avançants, ICREA, 08007 Barcelona, Spain; 3 Department of Ruminant Production, IRTA, 08140 Caldes de Montbui, Spain; 4 Blanca from the Pyrenees, Hostalets the Tost, 25795 Lleida, Spain; 5 Departamento de Mejora Genética Animal, Instituto Nacional de Investigación y Tecnología Agraria y Alimentaria O.A., M.P, 28040 Madrid, Spain; 6 Bioscience Research Division, ECODEV, Bundoora, 3038 Australia

Correction to: *Scientific Reports* 10.1038/s41598-018-36673-w, published online 09 January 2019

This Article contains a typographical error in the Methods section under subheading ‘Metagenome ensemble’ where,

“The annotation was performed with options –compliant –centre UoN –norrna –notrna –metagenome, and a bioproject was submitted to NCBI database with number PRJNA423102.”

should read:

“The annotation was performed with options –compliant –centre UoN –norrna –notrna –metagenome, and a bioproject was submitted to NCBI database with number PRJNA423103.”

