# Supplementary material for: Whole rumen metagenome sequencing allows classifying and predicting feed efficiency and intake levels in cattle
Source: Sci Rep. 2019 Jan 9;9:11. doi: 10.1038/s41598-018-36673-w (PMC6327033; doi:10.1038/s41598-018-36673-w)

Whole rumen metagenome sequencing allows classifying and predicting feed efficiency and intake levels in cattle

Beatriz Delgado[1], Alex Bach[2,3], Isabel Guasch[4], Carmen González[5], Guillermo Elcoso[4], Jennie E. Pryce[6], Oscar Gonzalez-Recio[1,5]

[1]Escuela Técnica Superior de Ingeniería Agronómica, Alimentaria y de Biosistemas. UPM. Ciudad Universitaria s/n 28040 Madrid, Spain

[2] Institució Catalana de Recerca i Estudis Avançants, ICREA, 08007 Barcelona, Spain.

[3] Department of Ruminant Production, IRTA, 08140 Caldes de Montbui, Spain.

[4] Blanca from the Pyrenees, 25795 Hostalets the Tost, Spain

[5] Departamento de Mejora Genética Animal. Instituto Nacional de Investigación y Tecnología Agraria y Alimentaria O.A., M.P. 28040 Madrid, Spain

[6] Bioscience Research Division, ECODEV, Bundoora 3038, Australia

Supplementary Table T1. ***Diet***. *Diet composition fed to BLANCA cows.*

Item	Amount
**Ingredient, % od DM**	
Fescue hay	22.46
Corn grain	20.55
Barley grain	19.15
Wheat silage	9.26
Soybean meal	8.85
Sunflower meal	4.26
Grass silage	3.31
Straw	3.27
Soybean hulls	2.90
Alfalfa hay	1.59
Molasses	1.50
Wheat middlings	0.73
Ryegrass hay	0.64
Calcium carbonate	0.50
Beet pulp	0.45
Sodium chloride	0.27
Magnesium oxide	0.18
Mineral vitamin premix	0.14
**Nutrient, DM basis**	
Crude protein, %	15.3
Neutral detergent fiber, %	34.9
Fat, %	2.45
Ash, %	5.66
Net energy of lactation, Mcal/kg	1.64

Supplementary Table T2. Summary of reads resulted from the metagenome sequencing of each sample*. Number of forward (R1) and reverse (R2) reads of each sample with their average length and corresponding standard deviation (SD).*

sample	R1reads	R1LenMean	R1LenSd	R2reads	R2LenMean	R2LenSd
S1	1211800	273.75	50.35	1211800	282.49	47.71
S2	1046062	278.98	44.87	1046062	287.24	41.02
S3	702149	279.99	46.36	702149	285.54	44.3
S4	1398741	282.96	43.98	1398741	288.21	41.27
S5	919530	286.24	39.63	919530	290.59	36.85
S6	1845909	270.82	57.54	1845909	278.49	55.84
S7	742156	250.29	65.16	742156	265.32	65.56
S8	1239730	276.48	46.72	1239730	286.05	42.6
S9	1024800	284.59	40.77	1024800	289.84	37.73
S10	1149620	280.6	43.83	1149620	287.61	40.52
S11	957466	277.11	28.65	957362	284.31	23.84
S12	848155	270.47	53.76	848155	279.52	51.76
S13	1165212	262.11	57.41	1165212	272.83	56.55
S14	679854	275.3	52.51	679854	282.48	50.2
S15	990621	275.04	49.18	990621	283.95	46.05
S16	887753	284.42	40.95	887753	289.55	38.06
S17	895359	276.49	51.96	895359	282.62	49.98
S18	1185485	274.22	50.64	1185485	283.39	47.5
S19	710081	283.08	41.85	710081	288.73	38.92
S20	1140761	275.08	49.05	1140761	283.04	46.62
S21	974114	279.88	47.93	974114	285.45	45.77
S22	795061	274.81	49.09	795061	282.78	46.79
S23	1128868	282.77	43.08	1128868	288.82	39.73
S24	1190329	278.78	50.85	1190329	284.26	48.8
S25	931155	286.68	38.2	931155	291.23	35.14
S26	1082971	287.97	35.81	1082971	292.3	32.61
S27	1461901	283.66	40.95	1461901	289.1	38.07
S28	878005	276.42	47.8	878005	284.28	44.93
S29	716606	242.6	66.81	716606	256.62	68.8
S30	2780620	286.69	37.81	2780620	291.33	34.71

Supplementary Figure S1. Microbiota variability*. Relative abundance of classified species for each individual.*

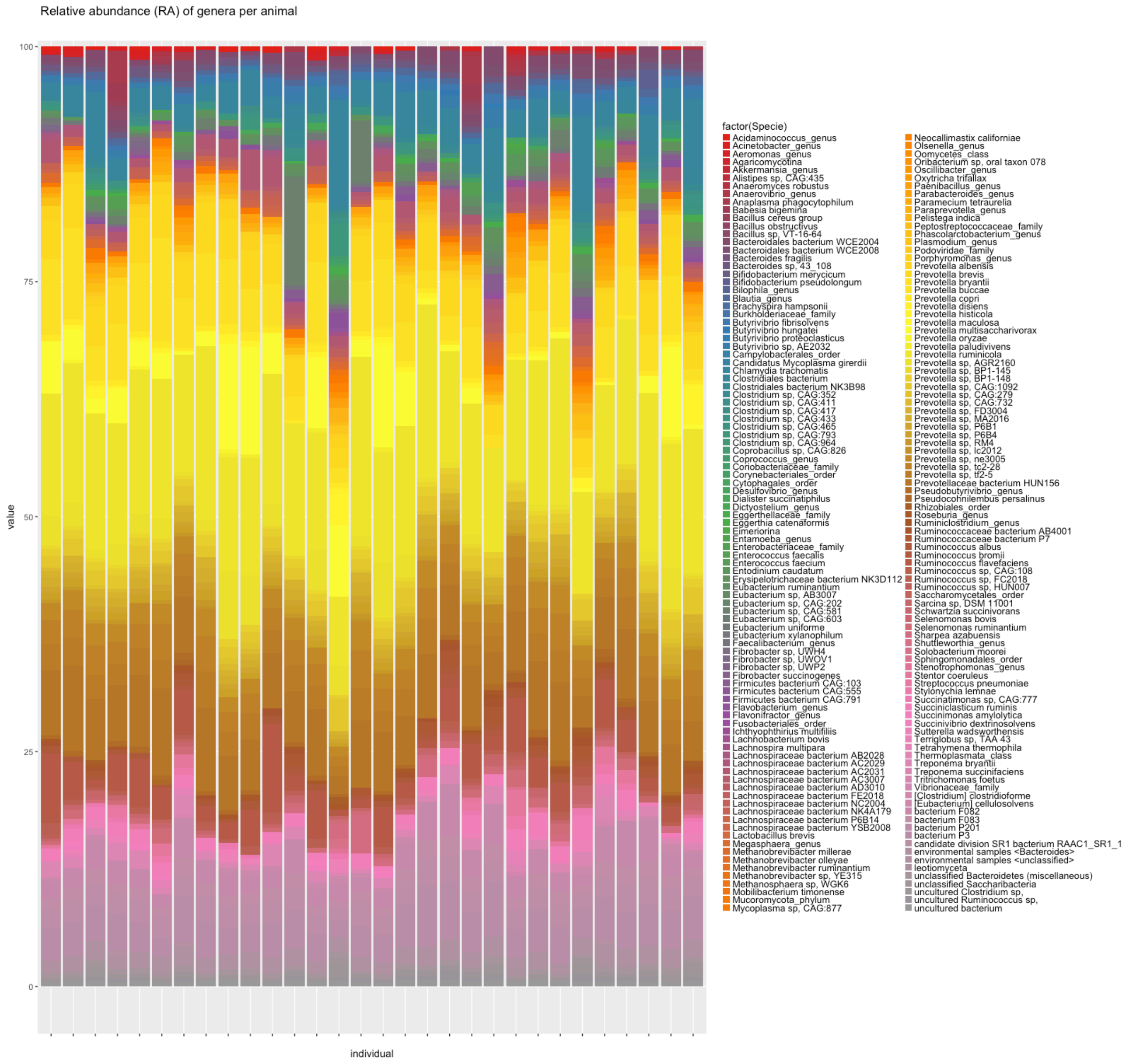

Supplementary Figure S2. Dry matter intake box plot. *Box plot for dry matter intake within feed efficiency group. Red numbers identify cows in the right-ended cluster in Figure 6b.*

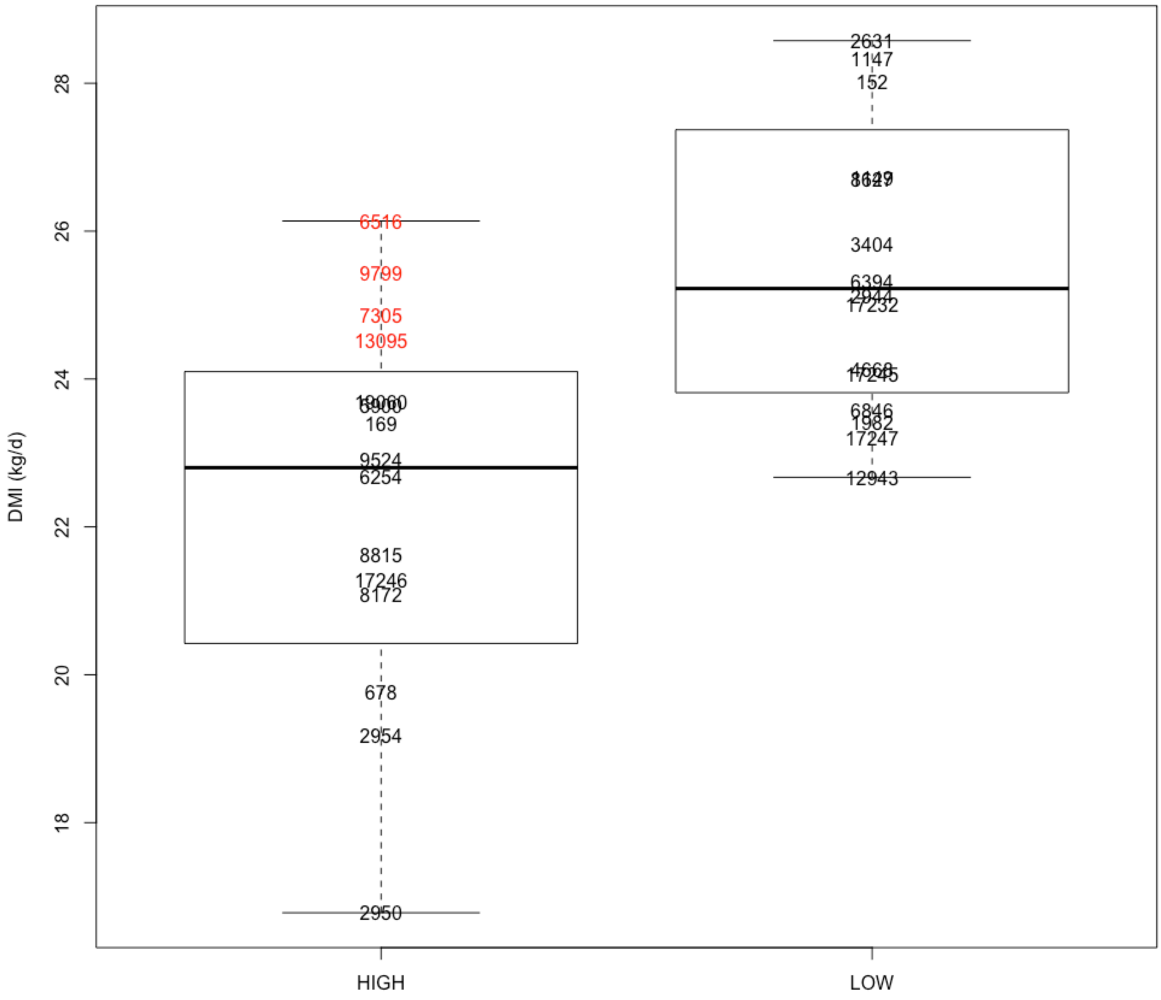

Supplement: Supplementary file 1 — Supplementary Info [file 41598_2018_36673_MOESM1_ESM.pdf]
